# More Than Just Records: Analysing Natural History Collections for Biodiversity Planning

**DOI:** 10.1371/journal.pone.0050346

**Published:** 2012-11-21

**Authors:** Darren F. Ward

**Affiliations:** New Zealand Arthropod Collection, Landcare Research, Auckland, New Zealand; University of Guelph, Canada

## Abstract

Natural History Collections (NHCs) play a central role as sources of data for biodiversity and conservation. Yet, few NHCs have examined whether the data they contain is adequately representative of local biodiversity. I examined over 15,000 databased records of Hymenoptera from 1435 locations across New Zealand collected over the past 90 years. These records are assessed in terms of their geographical, temporal, and environmental coverage across New Zealand. Results showed that the spatial coverage of records was significantly biased, with the top four areas contributing over 51% of all records. Temporal biases were also evident, with a large proportion (40%) of records collected within a short time period. The lack of repeat visits to specific locations indicated that the current set of NHC records would be of limited use for long-term ecological research. Consequently, analyses and interpretation of historical data, for example, shifts in community composition, would be limited. However, in general, NHC records provided good coverage of the diversity of New Zealand habitats and climatic environments, although fewer NHC records were represented at cooler temperatures (<5°C) and the highest rainfalls (>5000 mm/yr). Analyses of NHCs can be greatly enhanced by using simple techniques that examine collection records in terms of environmental and geographical space. NHCs that initiate a systematic sampling strategy will provide higher quality data for biodiversity research than ad hoc or point samples, as is currently the norm. Although NHCs provide a rich source of information they could be far better utilised in a range of large-scale ecological and conservation studies.

## Introduction

The 21st century offers a world of increasing connectance to biological information that is of direct relevance to biodiversity planning and conservation priorities [1]–[4]. It is widely acknowledged that Natural History Collections (NHCs) play a central role as sources of data for biodiversity and conservation [2], [4]–[9]. However, NHCs are also central to the interconnection of the ‘big new biology’ [3] in the 21st century because they are primary repositories of specimens and data. The ‘big new biology’ is the connection of taxonomic names with biological data/attributes occurring globally via the internet. This will enable biology to become more data-intensive by accommodating increasing amounts of data (e.g. molecular, large-scale digitisation projects from NHCs) and allowing biology to become more of a ‘big science’ [3].

NHCs collectively contain an estimated 2.5 billion specimens [4]. Specimens, and the information they contain, describe the identity and the temporal and spatial distributions of species. Consequently, NHCs, provide a massive source of data for a wide range of biodiversity and conservation studies [9]. However, despite their rich resources, NHCs have also been the subject of serious criticism, particularly for their inability to provide relevant information for 21^st^ century questions around the measuring and protecting of biodiversity. There are currently important issues around how these extensive collections can maintain their relevance to biological sciences [2], [5], [6], [8]–[10].

NHCs typically contain biological information that could be used for ecological questions on population sizes, the distribution of species, the number of species in an area, habitat associations, and the attributes of individual specimens [10]. However, the extent to which NHCs can provide information is often uncertain. Limitations include: the unknown sampling effort that was employed; the personal interests and curatorial techniques of collectors (e.g. discarding damaged individuals, only accessioning a certain number of individuals, targeting rare or unusual over common taxa); the spatial biases where areas have been under-sampled, or where samples are biased towards easily collected localities (e.g. near towns/cities and/or along roadsides); that information is often restricted only to the presence of a species (i.e. there is no information on where a species is absent); and the difficulty of getting information on other taxa from the same location (e.g. NHCs are organised taxonomically, not geographically).

However, this is not to say that NHCs and their data are not being used. Such data are indeed being used to address major themes in contemporary biological and ecological sciences, such as: spread of invasive species [11]–[13]; geographical patterns of environmental representation and diversity [6], [14], and climate change and other long-term temporal trends [9], [15], [16]. Yet there are, remarkably, very few quantitative reviews or historical analyses of NHC data that have examined biases within their holdings, and subsequently provided some recommendations on how to overcome these [6], [8].

In this paper, I examine a dataset of historical records of Hymenoptera to investigate spatial and temporal patterns of sampling in New Zealand. The dataset is used to examine how NHC records can contribute to several contemporary themes in biodiversity and conservation: invasive species, urbanisation, environmental representation, and long-term ecological research sites. I also use the dataset to illustrate how such analyses can inform the future management of NHCs in order to minimise, or avoid, biases in the future sampling of data, and be of even greater use to ecological and biodiversity sciences.

## Materials and Methods

### Study Area and Fauna

New Zealand comprises three main islands (North Island, South Island and Stewart Islands that span latitudes of 35–47°S, and have a cool to warm temperate climate with strong maritime and orographical influence [17].

**Figure 1:**
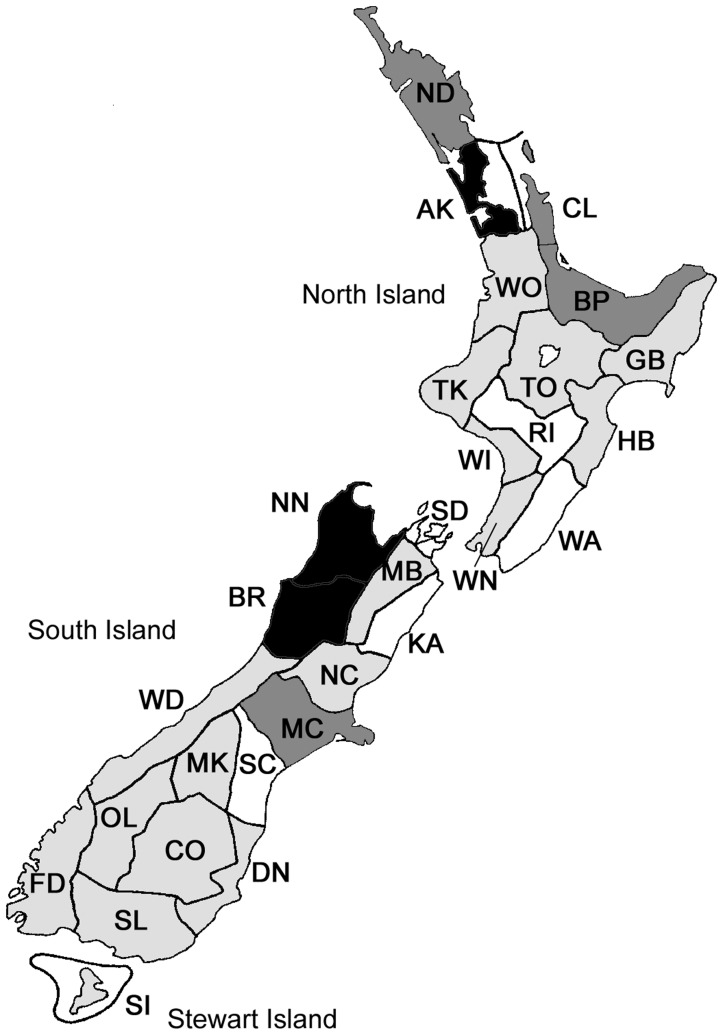
Summary of the spatial coverage ofNHC locations across New Zealand. Colour coding refers to the specific contribution of each area code [23] towards the overall Chi-square statistic (χ2 = 3573.27, df = 28, P<0.001). Very over-collected with >5% contribution (black), over-collected with <5% contribution (dark grey), under-collected with <5% contribution (light grey), very under-collected with >5% contribution (white).

The New Zealand Hymenoptera fauna is unusual [18]–[21], particularly for its near absence of sawflies [22], depauperate Aculeate fauna [18], and its very high diversity of Diapriidae and Mymaridae [23]. Species-level endemism is high (>90%) but there is an absence of many higher taxonomic levels [18]. For example, of the 90 or more families of Hymenoptera worldwide, only 46 are known in New Zealand. However, only 36 families are native; the other 10 families were either accidental introductions or deliberate introductions for biological control.

**Figure 2:**
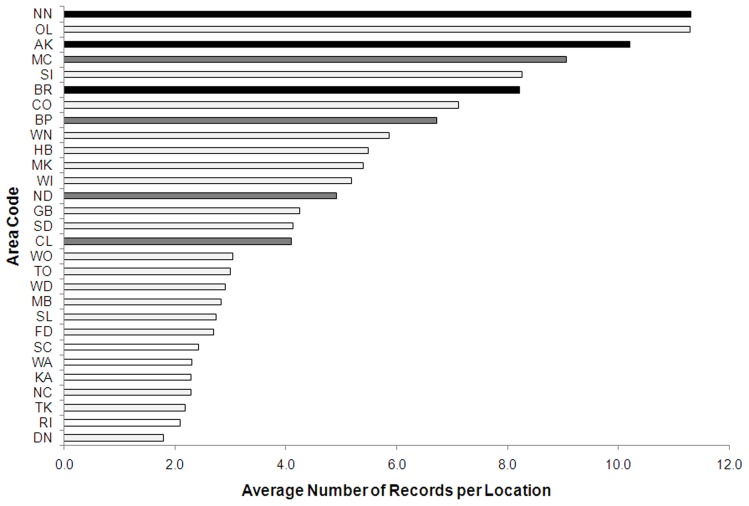
The average number of records per location from different area codes. Colour coding follows Figure 1.

**Figure 3:**
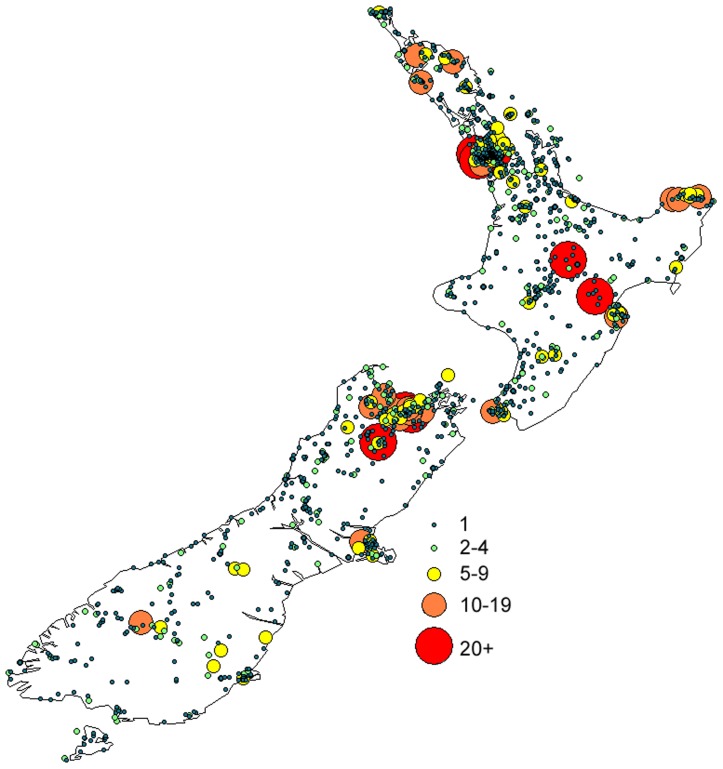
Sampling effort across New Zealand. Each location is marked by a circle, and the size of the circle represents repeated visits at a location (i.e. sampling effort).

### Collection Records and Taxonomic Groups

The New Zealand Arthropod Collection (NZAC) is the biggest holding of invertebrates in New Zealand (estimated >1 million pinned; 5–6 million ethanol). It was started in Nelson in 1920 as the Cawthron Institute collection, and is currently situated in Auckland at Landcare Research (see http://www.landcareresearch.co.nz/research/biosystematics/invertebrates/nzac).

**Figure 4:**
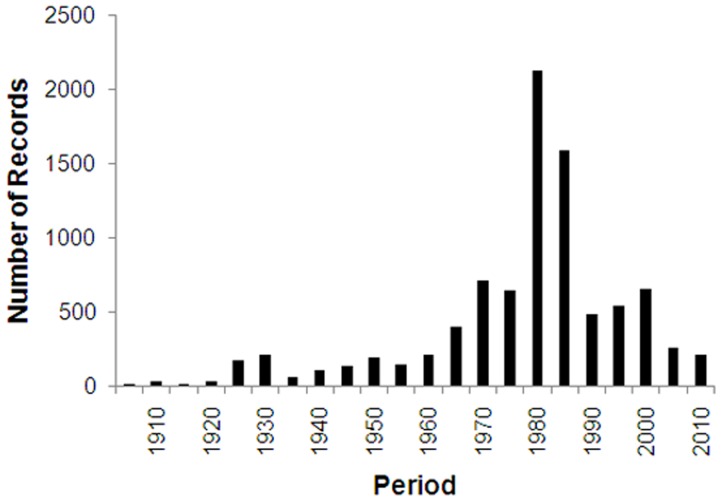
The number of NHC records at different time periods.

**Figure 5:**
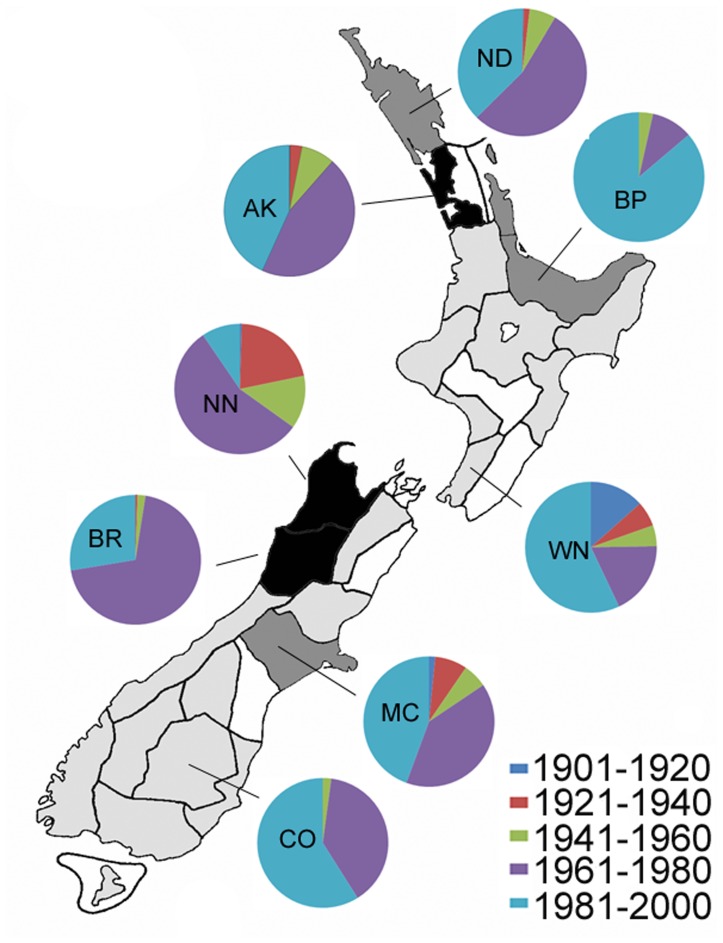
Proportions of NHC records collected at different time periods from selected area codes. Major proportions of NHC records have been collected at different time periods from different area codes.

The Hymenoptera section of the NZAC is estimated at ∼150,000 pinned specimens (2009 count), which is held in Cornell-style, glass-topped drawers. The Hymenoptera section is ordered taxonomically, with additional arrangement based on area codes. The New Zealand mainland (North, South, and Stewart Islands, plus nearby inshore islands) is subdivided into 29 approximately equal-sized areas, defined by two-letter “area codes” (Figure 1), and based on climatic areas used as weather forecast districts by the New Zealand Meteorological Service [24]. The main purpose of this system is to facilitate the arrangement, retrieval, and documentation of specimens in the New Zealand Arthropod Collection (NZAC).

**Figure 6:**
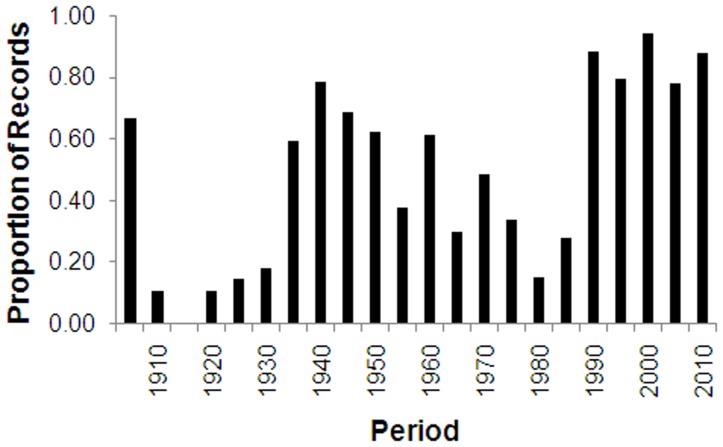
Proportion of records of introduced species from urban areas over time.

**Figure 7:**
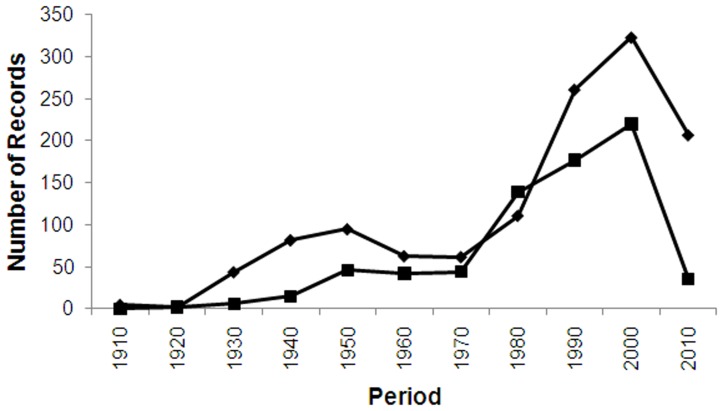
Number of records of introduced species from urban (diamond) and non-urban (square) locations.

Taxonomic groups used in the current paper belong to well-revised groups including major revisions of: Cheloninae (Braconidae) [25]; Pompilidae [26]; Ambositrinae (Diapriidae) [27]; Metopiinae (Ichneumonidae) [28]; Sphecidae (including Crabronidae) [29]; and Alysiinae (Braconidae) [30]. These groups have been completely databased and all NZAC records are used in the current paper. Other well-known groups in New Zealand that have been fully databased in the NZAC include Symphyta; Mutillidae; Scoliidae; Tryphoninae and Tersilochinae (both Ichenumonidae). Databased information on most Formicidae, and some Pteromalidae, Encrytidae [31], [32], and Mymaridae [33], are also used in the current paper. The composition of the dataset (compared with the total New Zealand Hymenopteran fauna) is slightly biased towards Aculeata and less towards Diapriidae and Chalcidoidea.

**Figure 8:**
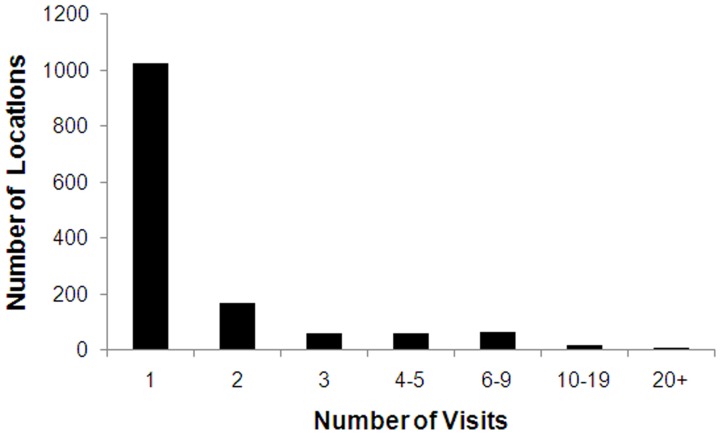
Frequency distribution of the number of repeat visits at specific locations. This indicates the number of locations with sufficient sampling effort that could potentially be used for long-term ecological research.

Databasing of the Hymenoptera section began in 2007, where information on specimen labels is digitised into a custom built database. Georeferenced points (decimal degrees) of sampling locations were either recorded at the time of sampling (from New Zealand Map Grid coordinates, or more recently using global positioning systems) or obtained retrospectively by matching site descriptions to georeferenced maps and databases (e.g. MapToaster Topo™).

**Figure 9:**
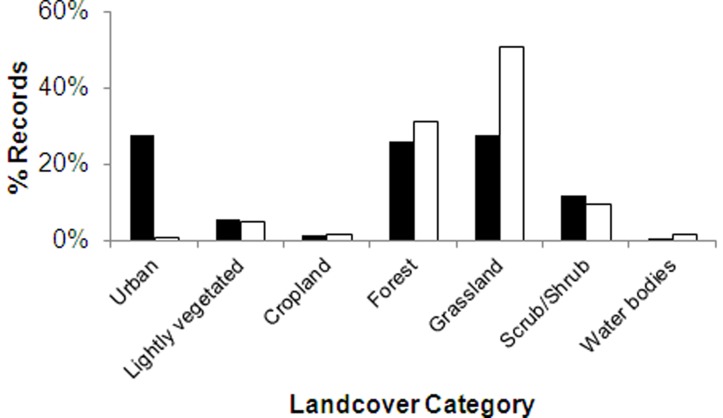
Comparison of NHC records (black) and background data (white) from different land-cover categories.

A location is spatially unique (i.e. separated from other “locations”), but a location can be visited at different times (e.g. months, years), contributing to sampling effort at that location, defined here as “records”. This is important as some locations have been visited more than others. Information from locations and records constitute the basic dataset on which analyses and discussion are based.

### Georeferenced Environmental Data

To determine whether NHC locations are representative of the overall New Zealand environment, 5000 random locations were generated across New Zealand to act as a background for comparison ( = “background data”).

**Figure 10:**
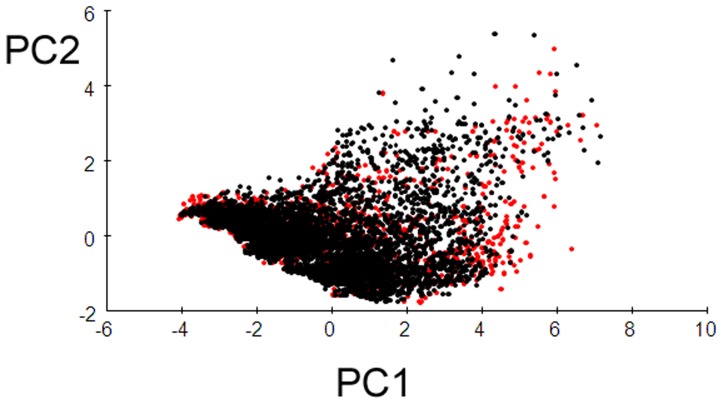
A principal components analysis (PCA) plot comparing NHC records (red) and background data (black). Eigenvectors are PC1 (mean annual temperature) and PC2 (mean annual rainfall).

**Figure 11:**
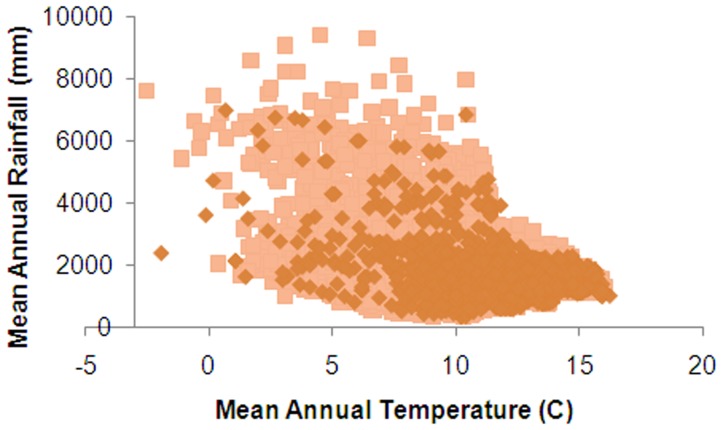
Comparison of NHC records (dark) and background data (light) for mean annual temperature and mean annual rainfall.

**Figure 12:**
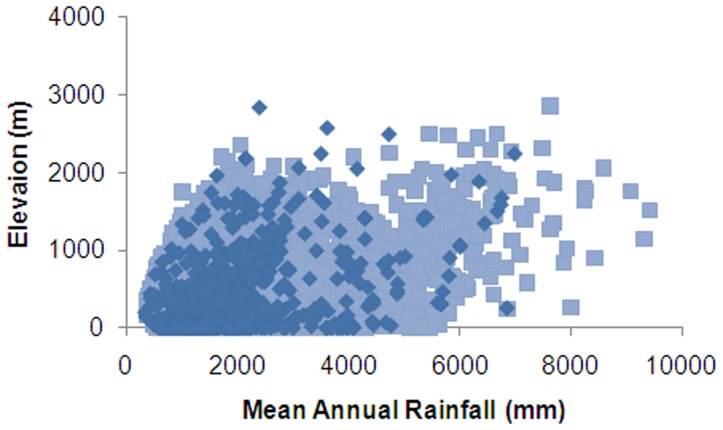
Comparison of NHC records (dark) and background data (light) for mean annual rainfall and elevation.

From the georeferenced locations (of NHC locations and background data), a number of environmental variables were obtained for each location: mean annual solar radiation (MAS, MJ/m2/day); mean annual temperature (MAT, °C); mean minimum daily temperature of the coldest month (TMIN, °C); mean annual rainfall (MAR, mm); degree growing days at 5°C (ggd5); and digital elevation (DEM, metres). Land-cover was obtained from the “Land Cover Database v2” (LCDB2), derived from satellite imagery [34]; and whether the location was within a protected national park, was also obtained.

**Figure 13:**
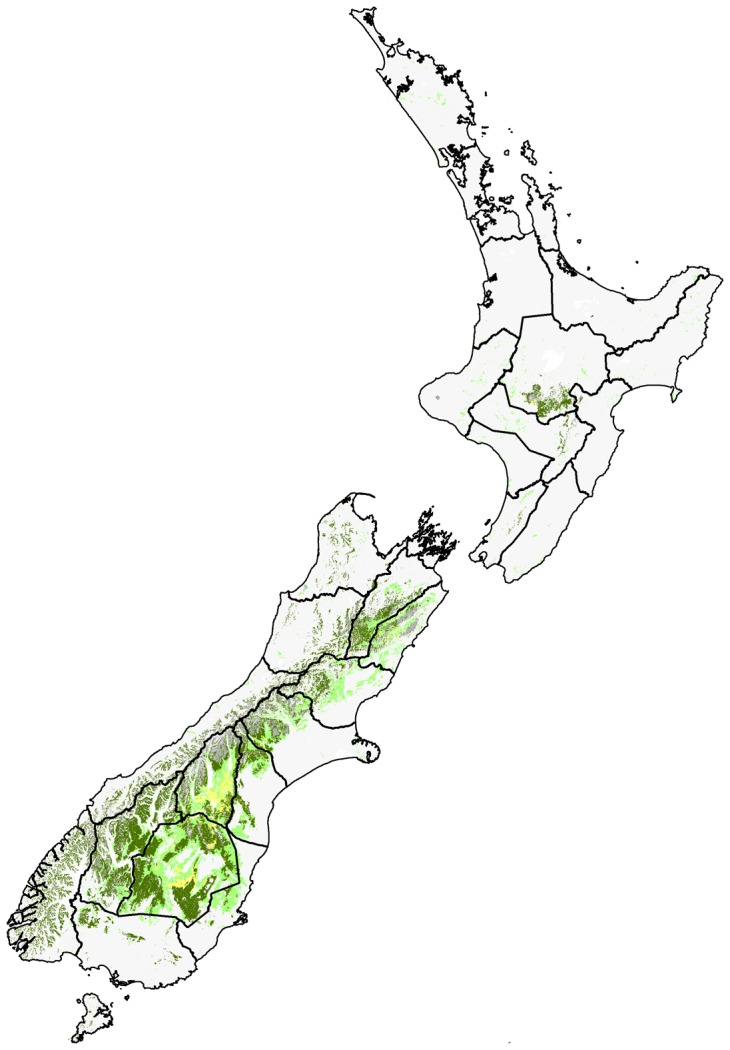
Extent of native habitats which are a priority for collection of Hymenoptera in New Zealand. Data based on further subdivision of LCDB2 native habitats, where the percentage difference between NHC and background records was greatest. Habitats are: Alpine Gravel and Rock (grey); Depleted Tussock Grassland (light yellow); Low Producing Grassland (light green); and Tall Tussock Grassland (dark green).

### Analyses

Locations were mapped in DIVA_GIS v5.0, a geographic information system designed for mapping and analysing biodiversity data (http://www.diva-gis.org/). Locations were categorised into area codes and analysed by Chi-square in Genstat® v8, with the option to examine the contribution of each category (i.e. area code) to the chi-square statistic. A principal component analysis (PCA) was used to examine NHC records in relation to background data in PRIMER v5.0 [35]. Contingency tables in Genstat® v8 were used to analyse NHC records associated with i) urban (vs non-urban) areas and introduced species, and ii) the number of records within a national park against background data. Urban environments were defined from LCDB2 categories as “Built-up Area”, “Transport Infrastructure” or “Urban Parkland/Open Space”.

## Results

### Spatial Coverage

The dataset contains information from 1435 locations across New Zealand, represented by >9300 NHC records and >15,000 specimens. The spatial coverage of locations was statistically uneven (χ2 = 3573.27, df = 28, P<0.001). Several area codes have been over-collected (codes = AK, BR, NN) and several under-collected (codes = RI, WA, KA, SC) (Figure 1). The same result was obtained whether analyses were conducted on either the number of specimens, the number of records, or the number of locations, because the different parameters linked to area codes were all very strongly correlated (average Pearson’s *r* = 0.996).

Area codes that have been significantly under-collected (codes = RI, WA, KA, SC) also have fewer records per location than over-collected area codes (Figure 2). When these records (i.e. the number of repeated visits to a location) are incorporated into spatial information, they show significant positive biases towards the top of the North, and the top of the South Islands (Figure 3). The top four area codes contributed >51% of records, and the top nine area codes contributed 75% of records.

### Temporal Coverage

The earliest records in the dataset are from 1900, representing some of the earliest collected Hymenoptera held in New Zealand (earlier collecting is generally held in overseas institutions). Over 40% of records were collected within a relatively small time period, between 1976 and 1985 (Figure 4), and trends in NHC records through different time periods are also evident over large spatial scales (Figure 5). Not surprisingly, field sampling has generally taken place in the Southern Hemisphere spring and summer periods, with 82% of records between October and March.

### Urbanisation and Introduced Species

NHC records from urban locations made up 31% of all records. Urban records were not evenly spread across area codes (Median % of urban records = 10%, range 0–59%), and several area codes had more urban records than non-urban records; AK (59% of all records), WI (54%), and DN (53%).

Urban areas were significantly associated with introduced species (Figures 6, 7). An introduced species was 5.6 times (χ2 odds ratio) more likely to come from an urban record than a non-urban record (Contingency table χ2 = 1198, P<0.001). However, NHC records also show that introduced species make up large extent of records for non-urban locations (Figure 7). Introduced species were mostly Aculeata (specifically Formicidae and Vespidae (61% records)), Ichneumonoidea (19%), Symphyta (9%) and Chalcidoidea (11%).

### Long-Term Ecological Research Sites

The most sampled location was “Nelson” with 129 collecting visits from 1920–1987. Nelson is a small city at the top of the South Island, were the NZAC first originated and also had a large institute for biological research, especially horticulture. Thus, it is not surprising it has many collection records. However, few other locations were as well sampled. Over 73% of the 1435 locations had only been visited once, and only 10 locations have more than 20 records (Figure 8). Repeated visits at just 2% of locations (n = 29) contribute 23% of all NHC records. In addition to the low number of visits per locations, many locations have their records spread over a long period but without a ‘core’ of records at certain periods to act as baseline data.

### Representation of Habitats and Climates

Comparison of land-cover categories between NHC records and the New Zealand background data confirmed the disproportionate amount of NHC records from urban areas, but also indicated some under-sampling of forest and grassland habitats (Figure 9).

The PCA showed a good visual overlap in environmental data for NHC records versus the New Zealand background data (Figure 10), although plots of environmental variables showed few NHC records were represented at cooler temperatures (<5°C) and highest rainfalls (>5000 mm) (Figures 11, 12). NHC records from national parks were in proportion to their extent nationally (Contingency table χ2 = 0.368, P<0.548). The first two eigenvectors of the PCA captured 84.5% of variation in environmental data. The largest coefficients were mean annual temperature (PC Eigenvector 1; 69.8% variation) and mean annual rainfall (PC Eigenvector 2; 14.7% variation).

## Discussion

Are collections data of any use for conservation and biodiversity decision making? [6]. Particular shortcomings of NHCs are often noted as being 1) geographically biased towards more easily accessed locations; 2) taxonomically incomplete, giving undue weight to a some taxa, 3) temporally biased, and 4) ad hoc in collecting effort. While it is widely acknowledged that NHCs are important sources of data for biodiversity and conservation [2], [4], [9], very few analyses of NHC data have examined biases within their holdings.

### Representation of the New Zealand Environment

The present study examined collection records (of Hymenoptera) to determine how well they represent the New Zealand environment. NHC records showed that the spatial coverage of locations was statistically uneven, with significant biases towards the lower latitudes in both the North, and South Islands. Furthermore, area codes with fewer locations sampled also had much lower sampling effort (repeat visits) in these locations. Temporal biases were also evident in the collection records, with a large proportion of records collected in a relatively small time period, between 1976 and 1985. This period coincides with high numbers of entomology staff across New Zealand institutes. It includes a project where many Malaise traps were operated throughout New Zealand to coincide with the visit of John Noyes (Natural History Museum, London) in 1980–1981 to work on Chalcidoidea [36]. Temporal biases also occurred at a smaller scale, where different area codes had different proportions of records from different time periods.

Surprisingly, NHC records from urban locations were disproportionally represented (31% of all records). However, some area codes had a greater proportion of urban records than others. Urban areas were also significantly associated with introduced species. An introduced species was 5.6 times more likely to come from an urban record than a non-urban record. These data most likely reflect research activities on introduced species in urban areas [12], [32] that, with their greater population density, have more people who, out of interest, collect and submit ‘bugs’ for identification that are deposited in NHCs. Many introduced species also initially establish in urban areas which are associated with trading ports [11].

Unfortunately, results from the current dataset indicate that NHC records would be of limited use for long-term ecological research, reflecting their inadequate temporal coverage. In addition to the low number of locations with a relatively good sampling history, some of the locations with good sampling history are i) ambiguous in their specific locality, for example, “Nelson”, or “Waitakere Ranges”, and/or ii) the records are spread sparsely over a long period (many decades), without a ‘core’ of records at certain periods. Such limited data compromise the ability to use historical data, especially analyses of shifts in community composition [15]. This can only be solved by the development of collection strategies over time for key sites.

Comparison of land-cover categories confirmed the disproportionate amount of NHC records from urban areas, but apart from this, NHC records in general provided good coverage of New Zealand habitats. There was also a high overlap between NHC records and background data for key climate variables. NHC records from protected national parks were in proportion to the area of protected national parks in New Zealand.

The current dataset of NHC records provides a relatively broad analysis from a national level over a 90-year period. A key question is whether the Hymenopteran dataset is representative of issues likely to occur across other groups of invertebrates? It may be that by using a greater number of records and/or a wider range of invertebrate taxa different patterns would emerge. Another possibility is that the collection data from the NZAC is not representative of all NHCs in New Zealand. The NZAC is by far the single biggest collection in New Zealand and its holdings and taxonomic coverage of Hymenoptera are the most extensive. However, other collections in New Zealand may provide a strong regional focus. At present, there is no way to determine if the patterns of NHC records would differ by incorporating further records, taxa, or other collections. However, different data sources frequently complement each other [8], so a combined dataset is likely to provide a more informative picture. In New Zealand (and elsewhere), the lack of a central database across all entomology collections is a significant issue, and needs urgent attention. There is a very strong need to link the holdings of these collections.

### Future Biodiversity Planning

The role of NHCs or biodiversity planning can be greatly enhanced by using simple GIS techniques that examine collection records in terms of environmental and geographical space [6], [8]. With the current dataset, several areas and habitats of New Zealand have now been identified as being under-represented in the national collection (NZAC). Through the use of GIS environmental layers, these areas and habitats can be visually mapped and future collection activities prioritised for them (see Figure 13). For example, several alpine and grassland habitats are identified as under-represented for Hymenoptera in New Zealand. Sampling effort could now be focused on the South Island alpine zone and, to a lesser degree, the North Island the central plateau (Figure 13).

Understanding patterns of biodiversity is a key aspect for conservation [6], [14]. Although collections data may not be perfect, they can assist biodiversity and conservation planning in several ways [6], [14], [15]. Using collections data in biodiversity studies adds value to the results and its importance should not be ignored. This may be especially important for invertebrates where incorporating invertebrate diversity into ‘mainstream’ conservation has been a major issue for several decades [37]–[40]. Although conservation programmes for individual rare species are important for public engagement, their scope is always limited [41], [42]. The use of indicator taxa and plant/vertebrate surrogates etc has also proved difficult [38], [39], [43]. However, the use of NHC records may prove a far better and easier way of including invertebrates into biodiversity and conservation planning. This type of data-driven research has largely been overlooked in the nexus between NHC records and biodiversity planning for invertebrates. Yet, data-intensive science is tailor-made for NHCs because of the large volumes of data from multiple sources and fields that are available [7].

I suggest priorities for NHCs include:

- **Mass databasing.** An important part of biodiversity planning is databasing. A recent publication summarised the importance of databasing, ‘records that are not georeferenced, dated, and fed into a centralised database have little future scientific value’ [8]. Databasing also helps secure historical data.
- **Analysis of holdings.** In order to maintain and increase their relevance for ecological and biodiversity sciences, NHCs need to take a self-critical look at their holdings, and in particular at how the specimen and data holdings are biased, what/where gaps exist, and how these could be managed for the future. The balance between infrequent collecting from many locations versus regular collecting at fewer locations is difficult to answer, but NHCs have generally neglected the latter option, and this should be somewhat ‘re-balanced’.
- **Identification of ecological datasets.** All NHCs will have specific datasets which are particularly valuable for ecological and biodiversity questions. Yet, almost all of these datasets will be unknown to ecologists/other interested researchers. For example, several datasets in the NZAC fall into this category. The “litter book” contains information of >4000 collection events to sample leaf litter from around New Zealand, and with specimens held in the NZAC. The data comes from many hundreds locations, across the 1966–1979 time period. Such data is an extremely valuable resource that could be used to examine land-use changes over time. Another project, could utilise a large collection of beetles from urban Auckland [44], to examine the effects of urbanisation on native species over time.
- **Repository for ecological projects.** Data from ecological surveys overcome many of the current limitations of the holdings of NHCs, that is, they record information on sampling effort, absence of species data, and populations sizes (or abundance). NHCs need to be open to the storage of material from ecological projects (be it well curated specimens, or bulk material). Although handling these projects can be time consuming for NHC staff. The fact is, such projects are exactly what is needed to answer many ecological questions that will arise in the future. If NHCs do not act as repositories, the biological material from these projects will be lost.

In summary, NHCs provide a rich source of information, however, they could be far better utilised in a range of large-scale ecological and conservation studies. In particular, NHCs must become drivers of biodiversity science.
